# Oral Microbial and Metabolic Alterations in Patients With Oral Lichen Planus Concomitant With Type 2 Diabetes Mellitus

**DOI:** 10.1002/mbo3.70361

**Published:** 2026-07-03

**Authors:** Xiaomeng Ren, Kaiyi Li, Xiaowen Kong, Jiawen Li, Hong Hua, Chunlei Li

**Affiliations:** ^1^ Department of Oral Medicine Peking University School and Hospital of Stomatology & National Center of Stomatology & National Clinical Research Center for Oral Diseases & National Engineering Research Center of Oral Biomaterials and Digital Medical Devices Beijing China; ^2^ Department of Oral Mucosa, Shanghai Stomatological Hospital Fudan University, Shanghai Key Laboratory of Craniomaxillofacial Development and Diseases Shanghai China

**Keywords:** disease severity, metabolomics, oral lichen planus, salivary flora, Type 2 diabetes mellitus

## Abstract

The present study aimed to comprehensively characterize the oral microbiome and metabolic profiles in patients with oral lichen planus (OLP) concomitant with type 2 diabetes mellitus (T2DM), and to explore the potential mechanisms driving the co‐occurrence. This was a cross‐sectional observational study. A total of 60 participants were enrolled, including 20 normal controls, 20 patients with OLP alone (OLP group), and 20 patients with both OLP and T2DM (OLP_DM group). Salivary samples were subjected to 16S rRNA sequencing and untargeted metabolomics to assess microbiological and metabolic differences across the groups. Spearman's correlation analysis was used to evaluate associations between clinical characteristics and microbial or metabolic features. Alpha diversity (Chao1 index) was significantly reduced in both disease groups compared to the controls, while beta diversity analysis revealed no remarkable separation among groups. At the genus level, the abundance of *Pseudomonas* was elevated in the OLP_DM group relative to both the OLP and control groups, and positively correlated with lesion severity. Metabolomic analysis revealed significantly lower levels of limonin and higher levels of thymine and epinephrine in the OLP_DM group compared to the OLP. Limonin was negatively correlated with lesion severity, whereas thymine and epinephrine showed positive correlations with both lesion severity and pain scores. The study provides comprehensive evidence of oral microbial dysbiosis and metabolic disturbances in patients with OLP concomitant with T2DM. The findings suggest an interplay between specific bacterial populations and metabolic alterations in the progression and severity of OLP concomitant with diabetes.

## Graphical abstract

We conducted a cross‐sectional study involving three groups: OLP‐DM, OLP, and healthy controls. Salivary 16S rRNA sequencing and untargeted metabolomics revealed distinct microbial and metabolic profiles among groups, which were associated with disease severity.

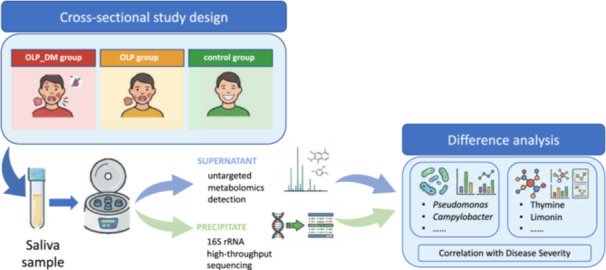

## 1 Introduction

Oral lichen planus (OLP) is a chronic inflammatory oral mucosal disease, with a prevalence rate from 0.81% to 0.98%. It is particularly common in middle‐aged women (Li 2020). Classified by the World Health Organization (WHO) as oral potentially malignant disorders, OLP carries a malignant transformation rate of 0.1%–3.2% (Gonzalez‐Moles and Ramos‐Garcia 2024; Offen and Allison 2022; Cheng 2022). Affected individuals often experience spontaneous pain and cancer‐related anxiety, leading to a substantial impact on both physical and mental well‐being of patients. The precise etiopathogenesis of OLP remains unclear. It is considered a multifactorial disease, with potential contributing factors including infections, genetic predispositions, and immune dysregulation. Recently, increasing attention has been directed towards the comorbidity of systematic diseases, particularly diabetes mellitus (DM), with OLP (Mallah 2021). Current management of OLP primarily relies on topical agents to alleviate symptoms and signs. Recently randomized controlled trials showed fluocinonide and tacrolimus reduced OLP severity (Polizzi 2025, 2023). However, these treatments remain palliative rather than curative, reflecting the fact that the underlying pathogenic mechanisms of OLP are yet to be fully elucidated.

DM, a widespread chronic metabolic disorder, affects over 800 million adults globally as of 2022, including approximately 148 million in China (Iacobucci 2024). The most prevalent form is type 2 DM (T2DM), is characterized by insulin resistance and chronic inflammation. The coexistence of OLP and DM (OLP_DM) was first reported by Grinspan in 1963, and studies indicate that diabetes is more common among OLP patients, with a pooled prevalence of 9.41% (95% CI = 8.16–10.74; OR = 1.64, 95% CI = 1.34–2.00, *p* < 0.001) (De Porras‐Carrique 2023). Clinically, OLP_DM patients tend to present with more severe clinical lesions at their initial visit compared to those with OLP alone, and they often require more frequent follow‐up appointments (Tenore 2025). Histopathologically, OLP_DM patients exhibit dense lymphocyte infiltration and significant inflammation at the basal layer, indicating a higher degree of tissue destruction (Kaiyi 2022). Risk models for OLP have identified diabetes as a significant risk factor, suggesting that better management of diabetes may lead to a marked improvement in OLP symptoms (Tar 2025). Therefore, the comorbidity of OLP and DM poses considerable challenges in clinical management and prognosis. The identification of specific biomarkers for both conditions, along with a deeper understanding of their comorbid pathogenesis, remains a critical focus for ongoing research in this field.

The oral microbiota is the second largest bacteria community in the human body. Its dysbiosis is closely linked to the progression of OLP, with enrichment of opportunistic pathogens such as *Prevotella* and *Fusobacterium* driving local inflammatory responses (Yan 2022; Du 2020; He 2017). Parallelly, DM has been shown to significantly alter the oral landscape; hyperglycemia not only reduces microbial richness but also promotes the growth of oral pathogens like *Porphyromonas gingivalis* (Li 2023). Beyond microbial shifts, the metabolic signature of saliva offers a window into these pathological processes. While specific biomarkers (e.g., (±)10‐HDoHE) and amino acid profiles have been identified for OLP and DM individually (Yan 2024), the comorbidity of OLP and DM remains a complex frontier. Given that elevated glucose levels and systemic metabolic dysfunction (such as altered branched‐chain amino acids (BCAA)) are hallmark features of DM (Roberts et al. 2014), they likely exert a unique selective pressure on the oral microenvironment. Consequently, we hypothesize that OLP‐DM comorbidity may exhibit a unique microbial and metabolic profile in saliva, which could influence the progression of OLP. However, this remains to be further elucidated. The present study aimed to comprehensively characterize the oral microbiome and metabolic profiles in patients with OLP and T2DM, and to explore the potential mechanisms driving the co‐occurrence.

## 2 Materials and Methods

This was a cross‐sectional observational study.

### 2.1 Ethics Statement

This study was approved by the Institutional Review Board of Peking University Hospital of Stomatology (Beijing, China) [PKUSSIRB‐201948109]. All participants provided informed consent before inclusion in the study and completed a basic questionnaire survey.

### 2.2 Study Subjects

Participants were recruited from individuals referred to the Department of Oral Medicine, Peking University Hospital of Stomatology, between September 1, 2020 and October 31, 2021. This study enrolled three groups of participants, patients with OLP alone (OLP group), patients with both OLP and T2DM (OLP_DM), and healthy individuals serving as controls.

**Inclusion criteria for OLP diagnosis:** Patients aged 18 to 75 years with a confirmed diagnosis of OLP according to the 2003 WHO clinical and histopathological criteria for OLP (van der Meij and van der Waal 2003). In our clinical practice, lesions associated with identifiable etiological factors (e.g. dental restorative materials or medications) were diagnosed as oral lichenoid lesions rather than OLP.

**Inclusion criteria for T2DM diagnosis:** T2DM was diagnosed in accordance with the 2019 WHO criteria (World Health Organization 2019), meeting any one of the following: (і) classic hyperglycemic symptoms (polyuria, polydipsia, polyphagia, unexplained weight loss) and random plasma glucose ≥ 11.1 mmol/L; (ii) fasting plasma glucose (FPG) ≥ 7.0 mmol/L (after ≥ 8 h of fasting); (iii) 2‐h plasma glucose during oral glucose tolerance test (OGTT) ≥ 11.1 mmol/L; (iv) glycated hemoglobin (HbA1c) ≥ 6.5% measured by standardized methods in certified laboratories with strict quality control.

**Inclusion criteria for the control group:** Adults aged 18 to 75 years with no oral mucosal diseases, no systemic diseases, and no history of chronic medication use.

**Exclusion criteria (applied to all groups):** The following exclusion criteria were applied: (і) pregnancy; (ii) presence of highly infectious diseases, other systemic conditions, or other precancerous lesions or malignant tumors; (iii) systemic diseases other than T2DM use of glucocorticoids or antibiotics within preceding 3 months; (iv) severe periodontitis (clinical attachment loss ≥ 5 mm, probing depth (PD) > 6 mm, and bone loss extending to the apical portion of the root), visible caries, or those using removable dentures or fixed orthodontic appliances.

### 2.3 Clinical and Laboratory Data Collection

Basic demographic and clinical data, including age, gender, smoking status, and general health conditions, were obtained through questionnaires. Body measurements, such as height, weight, and waist circumference, were recorded. The periodontal status of participants was assessed using community periodontal index (CPI). The subjective pain intensity associated with OLP was evaluated using the visual analog scale (VAS) (Faiz 2014). The severity of oral lesions was assessed by reticular/hyperkeratotic, erosive/erythematous, and ulcerative (REU) scoring system (Piboonniyom 2005). Additionally, FPG levels were measured for all participants.

### 2.4 Saliva Collection

Saliva samples were collected from resting, non‐irritating total saliva according to the guidelines of the Human Microbiome Project (USA) Participants were instructed to abstain from food and drink for at least 2 h prior to sampling, which was conducted between 8:00 a.m. and 11:00 a.m. Approximately 5 mL of saliva was collected in a sterile 50 mL conical tube. The samples were immediately refrigerated at 4°C and processed within 1 h by centrifugation at 12,000 g for 15 min. The supernatant and precipitation were stored separately at −80°C for subsequent untargeted metabolomics detection (supernatant) and 16S rRNA high‐throughput sequencing (precipitate).

### 2.5 Salivary Bacterial Community Analysis

Bacterial DNA was extracted from the saliva samples, followed by amplification of the V3‐V4 hypervariable regions of the bacterial 16S rRNA gene. Sequencing was performed on the Illumina MiSeq platform (Illumina, San Diego, USA). The sequencing reads were assigned to individual samples based on the unique barcodes. Paired‐end reads were pre‐processed using the Cutadapt software to remove adapter, and contaminating mitochondrial and chloroplast sequences were filtered out using the QIIME2 feature‐table plugin. Diversity metrics were calculated using the core‐diversity plugin within QIIME2, and the amplicon sequence variant (ASV) abundance table was generated. Alpha diversity indices, including Chao1, observed operational taxonomic units OTUs, Shannon index, Simpson index and Faith's Phylogenetic Diversity (Faith_PD), were estimate microbial diversity within individual samples. Beta diversity was measured using the Bray‐Curtis distance metric to assess microbial community variation across samples, and Permutational Multivariate Analysis of Variance (PERMANOVA) was used for statistical testing. Principal Coordinates Cnalysis (PCoA) was used for visualization. The Linear Discriminant Analysis Effect Size (LefSe) algorithm was employed to identify potential biomarkers for OLP, with a linear discriminant analysis (LDA) threshold was set as 3 (or 2.5 for OLP_DM compared to OLP).

### 2.6 Salivary Metabolomics Analysis

Metabolic profiling of saliva supernantat was performed using a Vanquish UHPLC system (ThermoFisher, Germany) coupled with an Orbitrap Q Exactive^TM^HF mass spectrometer (Thermo Fisher, Germany) at Novogene Co. Ltd. (Beijing, China). Raw data files generated by UHPLC‐MS/MS were processed using the Compound Discoverer 3.1 (Thermo Fisher) to peak alignment picking, and quantitation of each metabolite. Metabolites were annotated using the KEGG, HMDB database (https://hmdb.ca/metabolites) and LIPIDMaps (http://www.lipidmaps.org/) database. Orthogonal Partial Least Squares Discrimination Analysis (OPLS‐DA) was used to highlight the group differences. Differential metabolites were identified based on a fold change of ≥ 2 and a significance threshold of *p* < 0.05. Cross‐validation of differential metabolites was performed using the Random Forest algorithm.

### 2.7 Statistical Analysis

Given the exploratory nature of this multi‐omics study, the sample size was 20 participants per group to detect preliminary differences in microbial and metabolic profiles across groups. For normal distributed demographic and clinical data, *t*‐tests were employed for inter‐group comparison, and one‐way analysis of variance (ANOVA) was used for comparisons across multiple groups. For non‐normal distributed data, the Wilcoxon test was used for pairwise comparisons, and the Kruskal‐Wallis rank‐sum test was applied to multiple‐group comparisons. Spearman's correlation analysis was performed to assess relationships between indices. Statistical analyses were conducted using R software and GraphPad Prism, with SPSS 25.0 used for general statistical processing. All *p*‐values reported are two‐sided, with a significance threshold set as *p* < 0.05.

## 3 Results

### 3.1 Participants' Characteristics

A total of 60 participants were included in the study, consisting of 20 healthy controls (control), 20 patients with OLP alone (OLP), and 20 patients with OLP concomitant with T2DM (OLP_DM).

No significant differences were observed among the three groups with respect to gender, age, smoking status, height, weight, waist circumference, or periodontal health status (*p* ≥ 0.05). However, FPG levels were significantly higher in the OLP_DM group compared to the OLP group (*p* < 0.05). Additionally, compared to the OLP group, the OLP_DM group exhibited higher VAS scores and REU scores (Table 1).

**Table 1:** Demographic information and clinical feature of participants.

	Control group (*n* = 20)	OLP group (*n* = 20)	OLP_DM group (*n* = 20)	*p*
Males/Females	5/15	6/14	6/14	0.92
Age (M ± SD)	42.15 ± 6.72	44.40 ± 11.43	46.40 ± 8.15	0.32
Smoking (Yes/No)	3/17	2/18	5/15	0.44
Height (cm) (M ± SD)	163.80 ± 7.60	166.90 ± 7.12	164.60 ± 9.41	0.46
Weight (kg) (M ± SD)	62.90 ± 11.95	67.60 ± 11.27	67.95 ± 11.71	0.32
Waistline (cm) (M ± SD)	79.15 ± 8.24	82.25 ± 6.63	83.35 ± 7.14	0.18
FPG (mmol/L) (M ± SD)	5.07 ± 0.37^a^	5.12 ± 0.42^a^	7.49 ± 1.17^b^	< 0.001*
Periodontal health status				0.28
CPI = 0	3	2	2	
CPI = 1	6	6	6	
CPI = 2	6	5	7	
CPI = 3	5	7	5	
VAS (M ± SD)	NA	2.65 ± 0.88	3.30 ± 0.98	0.03*
REU (M ± SD)	NA	3.8 ± 1.08	6.25 ± 1.90	< 0.001*

*Note:* Values within the same row with different superscript letters (a, b) differ significantly; *p < 0.05.

Abbreviations: CPI, community periodontal index; FPG, fasting plasma glucose; M ± SD, mean ± standard deviation; NA, Not Applicable; OLP_DM, coexistence of OLP and type 2 diabetes mellitus; OLP, oral lichen planus; REU, reticular/hyperkeratotic, erosive/erythematous, and ulcerative; VAS, visual analog scale.

### 3.2 Overall Picture of Salivary Microbiota in the Three Groups

**Figure 1:**
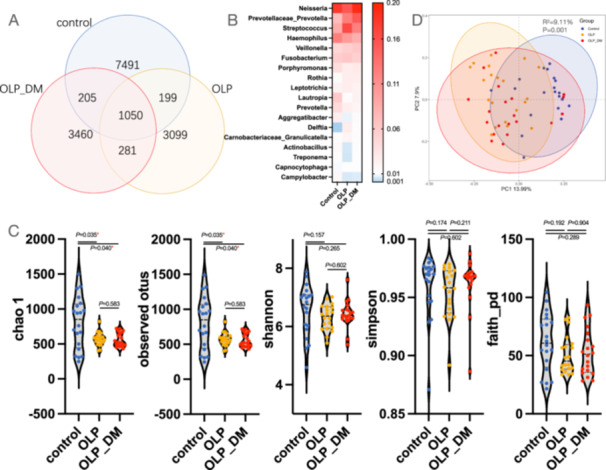
Overview of the microbiota composition in saliva samples from control, OLP and OLP_DM groups. (A) Venn diagram depicting the overlaps of ASVs (Amplicon Sequence Variants) among the three groups. (B) Proportional distribution of bacterial abundance at genus level across the groups. (C) Alpha diversity analysis showing the microbial richness and evenness in each group. (D) Principal coordinates analysis (PCoA) plot based on Bray Curtis dissimilarity, illustrating the microbial community structure across the three groups.

Following data processing, a total of 15,785 ASVs were identified, with 1,050 ASV being common across all three groups (Figure 1A). At genus level, 18 genera accounted for more than 1% of the relative abundance in at least one of the groups, collectively representing 82%–85% of the total microbial community (Figure 1B).

Alpha diversity analysis based on the Chao1 index revealed greater community richness in the control group compared to both the OLP and OLP_DM groups. However, no significant differences were observed among the groups in terms of Shannon index, Simpson index, or Faith_PD (Figure 1C).

PCoA using the Bray‐Curtis metric dissimilarity metric showed that samples from the control, OLP and OLP_DM groups clustered closely together, indicating relatively homogeneous microbial communities across groups (*R* ^2^ = 9.11%, *p* = 0.001) (Figure 1D).

### 3.3 Intergroup Variations in Microbial Communities

**Figure 2:**
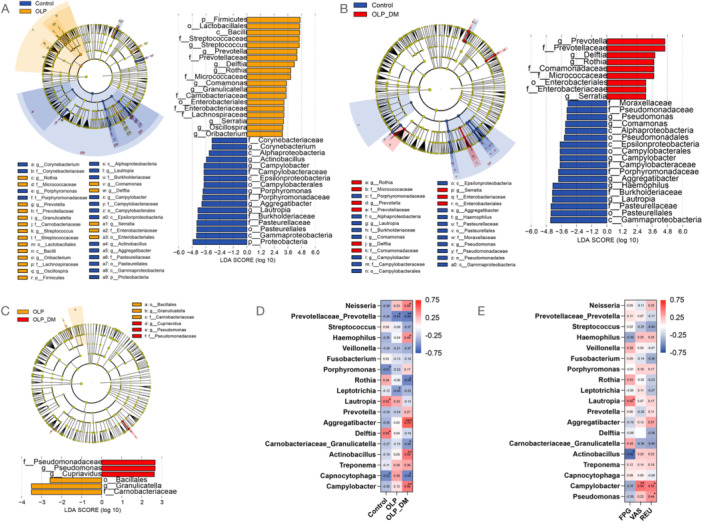
Relative abundance analysis of microbiota in saliva samples from control, OLP and OLP_DM groups. (A) Liner discriminant analysis (LDA) using LefSe algorithm comparing the OLP group with the control group. (B) LDA comparing the OLP_DM group with the control group. (C) LDA comparing the OLP group with the OLP_DM group. (D) Heatmap of Spearman correlation between *Pseudomonas* and the top abundant bacteria (relative abundance > 0.1%) in the three groups. (E) Heatmap of Spearman correlation between the top abundant bacteria and clinical features (FPG, VAS, REU).

At the phylum level, the OLP group exhibited a decreased abundance of Proteobacteria, while an enrichment of Firmicutes was observed. At the family level, significant reductions in the relative abundance of Burkholderiaceae, Campylobacteraceae, Pasteurellaceae, and Porphyromonadaceae were found in both OLP and OLP_DM groups compared to the controls. In contrast, the abundance of Enterobacteriaceae, Micrococcaceae and Prevotellaceae was higher in these groups. At the genus level, *Rothia*, *Prevotella*, *Delftia* and *Serratia* were more abundant in both OLP and OLP_DM groups compared to the controls, while *Lautropia*, *Campylobacter*, and *Aggregatibacter* were less abundant (Figure 2A,B). The OLP_DM group showed a higher relative abundance of the family Pseudomonadaceae (and its genus *Pseudomonas*) compared to both the OLP and control groups. Conversely, the OLP‐only group exhibited greater abundance of the family Carnobacteriaceae (and its genus *Granulicatella*) compared to the OLP_DM group (Figure 2C).

Further analysis was conducted to explore the correlation between the relative abundance of *Pseudomonas* and other key genera with abundance greater than 0.1% in each group. In both OLP and OLP_DM groups, the abundance of *Prevotella* (family Prevotellaceae) was negatively correlated with *Pseudomonas*. Additionally, the abundance of *Rothia*, *Capnocytophaga* and *Granulicatella* (family Carnobacteriaceae) in OLP_DM group also showed a negative correlation with *Pseudomonas*. In contrast, *Pseudomonas* abundance was positively correlated with *Nesseria*, *Haemophilus*, *Aggregatibacter*, *Actinobacillus* and *Campylobacter* (Figure 2D).

Focusing on the OLP_DM group, higher REU scores and higher VAS scores, indicative of more sever disease, were associated with a higher relative abundance of *Campylobacter*. Similarly, increased abundance of *Pseudomonas* was correlated with higher REU scores, suggesting a relationship between *Pseudomonas* and disease severity. However, neither of these genera showed a direct correlation with FPG levels (Figure 2E).

### 3.4 Metabolite Profiling and Description

**Figure 3:**
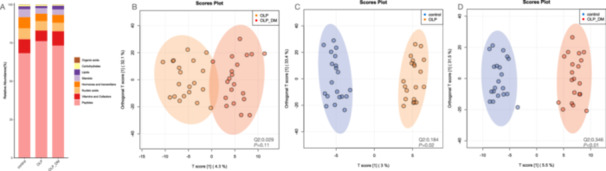
Overview of metabolite composition in saliva samples from control, OLP and OLP_DM groups. (A) Distribution of metabolites categorized by type in each group. (B) OPLS‐DA core plot comparing the OLP_DM and OLP groups. (C) OPLS‐DA core plot comparing the OLP and control groups. (D) OPLS‐DA core plot comparing the OLP_DM and control groups.

Categorized in three groups, peptides constituted the largest proportion, accounting for approximately 70% of the total metabolites. This was followed by vitamins and cofactors, which accounted for 9% in both control and OLP_DM groups, for 7% in OLP groups. (Figure 3A)

The OPLS‐DA models revealed considerable overlap in the metabolic profiles of the OLP and OLP_DM groups (Figure 3B). However, both the OLP and OLP_DM groups showed distinct metabolic patterns when compared to the normal control group (Figure 3C,D).

### 3.5 Metabolite Variations Across Groups

**Figure 4:**
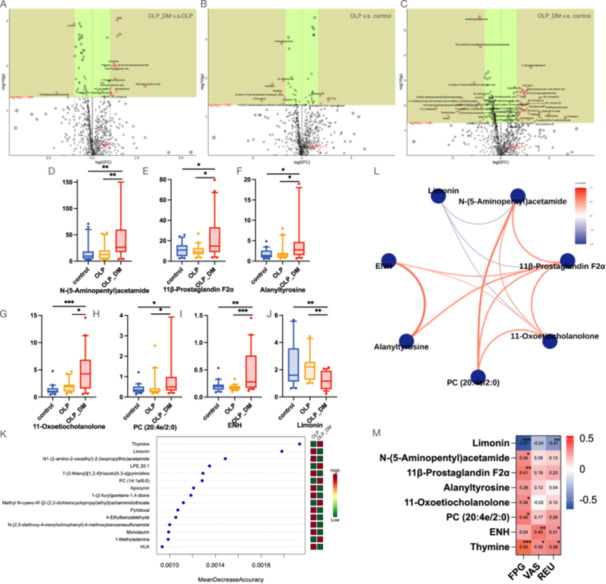
Metabolic differences among control, OLP and OLP_DM groups. (A) Volcano plot showing differential metabolites between the OLP_DM and OLP groups. (B) Volcano plot showing differential metabolites between the OLP and control groups. (C) Volcano plot showing differential metabolites between the OLP_DM and control groups. (D–J) Normalized abundance of representative differential metabolites. (K) Random forest analysis identifying differential metabolites between OLP_DM and OLP groups. (L) Network of representative differential metabolites, highlighting key associations. (M) Heatmap of Spearman correlation between representative differential metabolites and clinical features (FPG, VAS, REU).

Univariate analysis, visualized through volcano plots, highlighted group differences in metabolite abundance. 14 kinds of metabolites were more abundant in the OLP_DM compared to OLP, while 2 metabolites were more abundant in OLP than in OLP_DM. Additionally, 31 metabolites were enriched in the OLP_DM group relative to the control group, while 14 metabolites were less abundant (Figure 4A–C). Figure 4D–J provide individual visualizations of the normalized abundance of representative differential metabolites.

Random forest analysis was conducted to identify differential metabolites between the OLP_DM and OLP groups. Thymine was significantly more abundant in the OLP_DM group than in the OLP only group, with the highest mean decrease accuracy (Figure 4K). On the other hand, Limonin was markedly less abundant in patients with OLP_DM compared to both the OLP and control groups. As limonin level decreased, the abundance of LSD‐d3, N‐(5‐aminopentyl) acetamide, 11β‐prostaglandin F2α, and 11‐oxoetiocholanolone increased (Figure 4L).

In both OLP and OLP_DM samples, the abundance of ENH (Epinephrine Nitrate/Hydrochloride) was positively correlated with both VAS and REU scores, but not with FPG. Thymine abundance was also positively correlated with higher VAS and REU scores, as well as with FPG. Conversely, lower abundance of limonin was associated with higher REU scores and FPG levels (Figure 4M).

## 4 Discussion

To our knowledge, this study was the first to characterize the oral microbiome and metabolic profiles in patients with OLP concomitant with T2DM. We found that this comorbid condition was associated with reduced oral microbial alpha diversity, a marked enrichment of *Pseudomonas*, and distinct alterations in the level of thymine, epinephrine (ENH), and limonin, all of which correlated with disease severity. These findings suggest a potential interplay between oral microbial dysbiosis and metabolic disturbances in the pathogenesis of OLP_DM comorbidity.

The results revealed that both the diversity and abundance of salivary microorganisms were lower in the OLP and OLP_DM groups compared to the control group. This finding is consistent with previous studies, which reported a significant reduction in the diversity of the salivary microbiota in patients with OLP or those in a hyperglycemic state compared to healthy controls (Wang 2015; Goodson 2017). A reduction in oral bacterial diversity has been associated with an increased risk of oral mucosal disease. It is suggested that alterations in the microbiota disrupt the equilibrium of the microbial community, compromising the epithelial barrier and facilitating the entry of pathogens (Freire et al. 2021).

Further analysis at genus level revealed that *Rothia*, *Prevotella*, *Delftia* and *Serratia* were significantly enriched in patients with OLP, regardless of DM comorbidity, compared to the control group. Similarly, *Prevotella* and *Rothia* have been reported to be more abundant in patients with OLP compared to the healthy individuals (Wang 2015; Deng 2020).

Our findings highlighted a significant microbial shift in the OLP_DM group, characterized by the enrichment of *Pseudomonas* and its strong co‐occurrence with *Campylobacter*. Notably, both genera exhibited a significant positive correlation with the clinical severity of OLP, suggesting their synergistic role in driving disease progression. While other taxa such as *Neisseria* and *Haemophilus* also positively correlated with *Pseudomonas*, their lack of association with clinical scores suggests they may represent a restructured ecological background that facilitates the colonization of more aggressive pathogens under diabetic conditions. Previous studies have similarly identified an enrichment of *Pseudomonas* in the subgingival plaque of erosive OLP patients (Liu 2021). As an opportunistic pathogen, *Pseudomonas* can upregulate virulence factors, such as proteases, in response to high glucose levels and the chronic inflammatory state of OLP_DM (Arfaoui 2024; Suresh et al. 2023). These factors can degrade tight junction proteins and compromise the epithelial barrier (Golovkine et al. 2017), thereby increasing the penetration of pro‐inflammatory mediators and exacerbating the T‐cell‐mediated immune response in the underlying tissues.

Our metabolomic profiling revealed a distinct metabolic signature in the saliva of patients with OLP_DM, characterized by a significant depletion of protective metabolites and a concomitant elevation of stress‐related markers. Notably, the abundance of limonin was found to be significantly reduced in the OLP_DM group compared to both OLP and healthy controls, showing a strong negative correlation with disease severity and FPG levels. Limonin has emerged as a multifunctional protective agent; Previous studies underscore limonin's pharmacological potency in blunting postprandial glucose excursions. By competitively antagonizing α‐glucosidase, limonin effectively decelerates the hydrolytic cleavage of complex carbohydrates, thereby buffering the systemic circulation against rapid glycemic surges (El‐Feky et al. 2024). Furthermore, limonin has been shown to enhance insulin sensitivity by modulating the IRS‐1/GLUT4 signaling axis, effectively lowering blood glucose in metabolic syndrome rat model (Maneesai 2023). Beyond its metabolic benefits, the anti‐inflammatory and antioxidant properties of limonin may play a crucial role in maintaining oral mucosal homeostasis. It is known to suppress macrophage infiltration and down‐regulate key pro‐inflammatory cytokines, including IL‐6, IL‐1β, and TNF‐α (Li 2021). In keratinocytes stimulated by TNF‐α/IFN‐γ, limonin has demonstrated the capacity to inhibit MAPK signaling, a pathway frequently implicated in the pathogenesis of chronic inflammatory skin and mucosal diseases (Jin 2024). Moreover, limonin prevents mitochondrial dysfunction in inflamed epithelial cells by scavenging mitochondrial reactive oxygen species (ROS) (Lee 2024). Given that elevated TNF‐α and oxidative stress are hallmarks of OLP, our findings suggest that a deficiency in salivary limonin may eliminate a critical defensive barrier, thereby linking hyperglycemia to the accelerated progression of OLP lesions.

Our findings demonstrated that salivary epinephrine (ENH) and thymine levels were not only significantly elevated in the OLP_DM group but also exhibited a strong positive correlation with disease severity. Furthermore, thymine levels demonstrated a positive correlation with FPG concentrations. These findings suggest that both ENH and thymine may serve as active participants in, or predictive markers for, the clinical progression of OLP. The accumulation of thymine appears to be a distinct feature of OLP combine with DM, for its level decreased in patients with periodontitis and dental caries compared with healthy control (Yama 2023). This abnormal increased level of thymine may be driven by hyperglycemia‐induced ROS production causing DNA damage in mucosal cells, combined with the accelerated turnover and lysis of oral microbiota (such as Pseudomonas) in the glucose‐rich environment. The elevation of ENH may reflect sustained local catecholamine stress, which has been shown to exert cytotoxicity by activating the STAT3 signaling pathway and inducing oxidative stress in keratinocytes (Kim 2025). This process likely exacerbates mucosal damage under the systemic metabolic stress of diabetes. In conclusion, the synergistic effect of glucose‐driven oxidative pressure and stress‐induced cytotoxicity destabilizes the oral mucosa. These factors act in concert to promote the clinical progression of OLP.

We should acknowledge several limitations in our study. First, while 16S rRNA sequencing effectively profiles microbial communities at the genus level, it lacks the resolution for species‐level identification, necessitating. Future studies should consider metagenomic sequencing or targeted culturing. Approaches to achieve more precise taxonomic characterization. Second, the cross‐sectional design of this study precluded causal inference and limits the possibility to capture the longitudinal dynamics of the identified biomarkers. Future longitudinal studies are warranted to determine whether the observed microbial and metabolic alterations truly drive the pathogenesis of OLP_DM comorbidity, and it may serve as targets for future therapeutic exploration.

## 5 Conclusion

In summary, our study suggests that the increase in the abundance of *Pseudomonas* and decrease in the limonin levels could play significant roles in the onset or progression of OLP, especially in the presence of comorbid DM. These findings highlight a potential microbial‐metabolite interplay that could modulate host inflammatory pathways, thereby exacerbating disease severity.

## Author Contributions

**Xiaomeng Ren:** writing – original draft, formal analysis, visualization, investigation, validation, software. **Kaiyi Li:** investigation, formal analysis, data curation, project administration, software. **Xiaowen Kong:** investigation. **Jiawen Li:** investigation. **Hong Hua:** conceptualization, writing – review and editing, methodology, supervision, resources. **Chunlei Li:** writing – review and editing, conceptualization, methodology, funding acquisition, resources, supervision.

## Ethics Statement

This study was approved by the Institutional Review Board of Peking University Hospital of Stomatology (Beijing, China) [PKUSSIRB‐201948109].

## Conflicts of Interest

None declared.

## Data Availability

The data that support the findings of this study are available on request from the corresponding author. The data are not publicly available due to privacy or ethical restrictions.
